# Plasticity of the Primary Motor Cortex in Patients with Primary Brain Tumors

**DOI:** 10.1155/2020/3648517

**Published:** 2020-07-03

**Authors:** Nathan W. Kong, William R. Gibb, Suvarna Badhe, Benjamin P. Liu, Matthew C. Tate

**Affiliations:** ^1^Department of Medicine, University of Chicago, Pritzker School of Medicine, Chicago, IL, USA; ^2^Department of Emergency Medicine, Stanford University, Palo Alto, CA, USA; ^3^Department of Radiology, Northwestern University Feinberg School of Medicine, Chicago, IL, USA; ^4^Departments of Neurological Surgery and Neurology, Northwestern University Feinberg School of Medicine, Chicago IL, USA

## Abstract

There are two neuron-level mechanisms proposed to underlie neural plasticity: recruiting neurons nearby to support the lost function (ipsilesional plasticity) and uncovering latent pathways that can assume the function that was lost (contralesional plasticity). While both patterns have been demonstrated in patient groups following injury, the specific mechanisms underlying each mode of plasticity are poorly understood. In a retrospective case series of 13 patients, we utilize a novel paradigm that analyzes serial fMRI scans in patients harboring intrinsic brain tumors that vary in location and growth kinetics to better understand the mechanisms underlying these two modes of plasticity in the human primary motor cortex. Twelve patients in our series had some degree of primary motor cortex plasticity, an area previously thought to have limited plasticity. Patients harboring smaller lesions with slower growth kinetics and increasing distance from the primary motor region demonstrated recruitment of ipsilateral motor regions. Conversely, larger, faster-growing lesions in close proximity to the primary motor region were associated with activation of the contralesional primary motor cortex, along with increased activation of the supplementary motor area. These data increase our understanding of the adaptive abilities of the brain and may lead to improved treatment strategies for those suffering from motor loss secondary to brain injuries.

## 1. Introduction

Plasticity refers to the ability of the brain to reorganize its functional networks during normal development as well as to preserve function during times of stress or pathology. Brain plasticity has been demonstrated in a variety of brain regions and injury contexts, most notably ischemic stroke and brain tumors [1–9]. A variety of experimental tools have been used to measure plasticity in humans, including direct electrical cortical stimulation, transcranial magnetic stimulation, and functional magnetic resonance imaging (fMRI). Of these techniques, fMRI has been most extensively utilized given its ability to simultaneously interrogate multiple functional brain regions in a noninvasive manner. Both language plasticity and motor plasticity have been demonstrated using fMRI in patients with brain lesions [1, 5–11].

While there is evidence of plasticity in the human cortex, there is no clear consensus on the mechanisms through which this plasticity occurs. In a broad sense, there are two ways in which plasticity can occur. First, healthy synapses can be recruited from the region immediately around a lesion. This mode of ipsilesional plasticity has been demonstrated in both traumatic brain injury and spinal cord injury patients [12, 13]. A second mechanism relies on redundant connections that are typically silenced in the healthy brain. Upon injury, these redundant synapses can be called upon to restore function. This recruitment of dormant connections has been reported in patients with strokes, gliomas, and arteriovenous malformations [7, 14]. To this point, the most specific model for neural plasticity that has been proposed in humans is the interhemispheric inhibition theory. The foundational principle of this theory is that motor activation in one hemisphere suppresses activation of the corresponding motor cortex in the contralateral hemisphere [15]. Thus, injury in one hemisphere will release the inhibition of the contralateral hemisphere and allow it to take over function that is lost. This theory assumes that the motor cortex has bilateral projections to peripheral muscles, a finding demonstrated in primates and speculated to be present in humans [16]. Since this theory was first reported, there has been extensive work in patients with strokes trying to elucidate the precise mechanism in humans [17, 18], with mixed results, possibly due to overwhelming of plasticity mechanisms from the immediate and often large territory of neural injury seen in ischemic stroke, which is the injury type most extensively investigated in humans. In terms of studying plasticity, brain tumors, in contrast to ischemic stroke injuries, are more heterogeneously distributed and grow at varying rates, thus offering the advantage of a more dynamic insight into plasticity mechanisms.

In the present study, we evaluate plasticity of the hand region of the human primary motor cortex using serial fMRI evaluations in patients harboring intrinsic brain tumors of varying growth velocities and brain locations. Novel findings include a high degree of observed plasticity in our cohort and demonstration of both ipsilesional and contralesional patterns of plasticity as well as increased activity in the supplementary motor area. Finally, we report on patient- and tumor-associated factors that are associated with each pattern of plasticity. These data may provide additional insight into the functional anatomy of brain reorganization following injury.

## 2. Material and Methods

### 2.1. Patient Selection and Demographics

**Figure 1:**
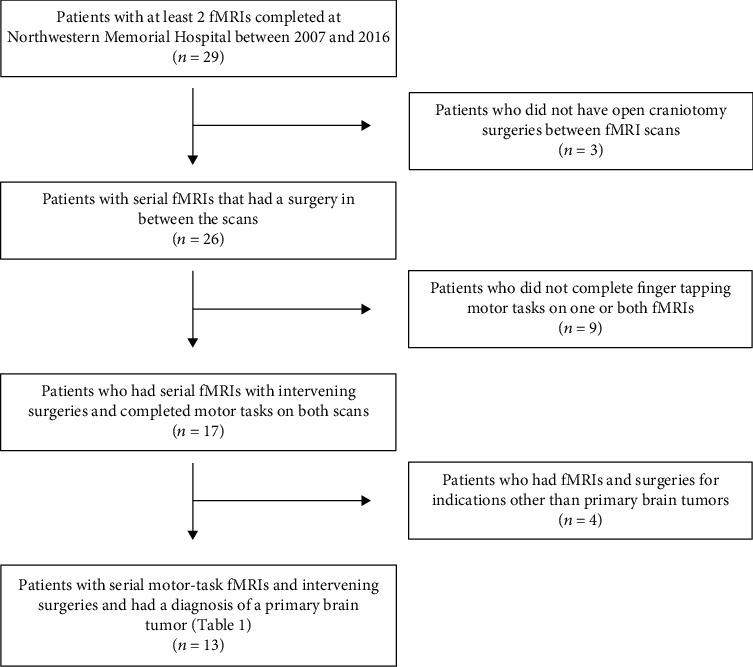
Selection of patients. Flowchart of patient selection for the present study.

All adult patients with primary intrinsic brain tumors (gliomas) from January 1, 2007, through December 31, 2016, who had two fMRI scans with finger-tapping motor tasks performed at Northwestern Memorial Hospital and had a surgical resection of the glioma between the two scans were eligible for inclusion (Figure 1). The institutional review board at Northwestern University reviewed and approved this study.

Patient data was collected using the electronic health record. The time between scans was recorded as the number of months between the first fMRI scan and the second fMRI scan. Pathologic grade was determined by a certified neuropathologist based on intraoperative tissue sampling. The volume of the enhancing lesion was determined using Brainlab Elements software (Brainlab AG, Feldkirchen, Germany) on T1 MPRAGE postcontrast weighted MRI images. The average growth velocity of the tumor was calculated as the difference in volumes between the first and second fMRIs divided by the time between scans in months. Distance from the motor cortex was calculated as the shortest distance from the edge of a hyperintense FLAIR signal to the center of the hand activation as seen on fMRI. We used the Neurologic Assessment in Neuro-Oncology (NANO) scale to quantify the degree of strength for each patient on the clinical exam [19]. The score for each patient reflects the strength in the weakest limb based on either a neurologist or a neurosurgeon's clinical exam prior to the surgery. Strength is scored on a scale from 0 (normal movement) to 3 (no movement).

### 2.2. fMRI Acquisition and Analysis

fMRI was acquired twice in all subjects using a hand motor task paradigm for analysis. A finger-tapping task was utilized using a block motor pattern beginning with 20 seconds of rest (A) and alternating 20 seconds of right-handed finger tapping (B) and 20 seconds of left-handed finger tapping (C) for a design of A_1_B_1_C_1_A_1_B_2_C_2_A_3_B_3_C_3_. Prior to the fMRI, the patients were trained to ensure proper performance of the task, and during the task, a screen with visual cues instructed the subjects when to move or stop movement. Patients were asked to touch fingers to thumb on each hand to a goal rate of 60-120 beats per minute. During scanning, patients were monitored in real time to ensure adequate motor activation was achieved. All patients were able to complete the tasks adequately. All fMRIs were scanned on a 3-Tesla MRI system (Siemens MAGNETOM Verio/Skyra, Munich, Germany) with the following scan parameters: fMRIs done after November 2013 were using a 12-element head coil, MDDW echo planar imaging sequence, TR 2000 ms, TE 20 ms, EPI factor 120, field of view 220 mm, 3 mm sections, 31 slices, matrix 120 × 128, voxel size 1.7 × 1.7 × 3.0 mm, transversal orientation, 120 measurements, 90-degree flip angle, bandwidth of 1446 Hz/Px, and echo spacing of 0.78 msec. fMRI scans done before November 2013 used ep2D blood-oxygen-level- (BOLD-) dependent imaging sequence, TR 2190 ms, TE 20 ms, EPI factor 120, field of view 220 mm, 3 mm sections, 32 slices, matrix 120 × 128, voxel size 1.7 × 1.7 × 3.0 mm, transversal orientation, 240 measurements, 90-degree flip angle, bandwidth of 1502 Hz/Px, and echo spacing of 0.75 ms. The change in MRI parameters was only applicable to two patients.

fMRI data was transferred to a separate workstation for analysis. Sequences were normalized spatially to the Montreal Neurologic Institute (MNI) 152 template brain space [20]. Nonbrain structures were removed from the T1 images using the brain extraction tool (BET) of the FMRIB software library (FSL; http://http://www.fmrib.ox.ac.uk/fsl) [21]. Lesion masks were manually applied to signal on FLAIR-sequenced images. Registration was performed using FSL's linear image registration tool (FLIRT) as described elsewhere and with motion correction using MCFLIRT [22, 23]. fMRI data processing was carried out using FSL's fMRI expert analysis tool (FEAT). Spatial smoothing (Gaussian kernel, FWHM 6.0 mm) and high temporal filtering (Gaussian-weighted least-squares straight line fitting, with stigma = 45.0 s) were used. Time-series statistical analysis was completed using FMRIB's Improved Linear Model (FILM) with local autocorrelation correction [24]. *Z* statistic images were thresholded using clusters determined by >50% local maximal activation and a cluster significance threshold of *p* = 0.001 [25, 26]. Region of interest (ROI) analysis was carried out using FSL's featquery tool.

### 2.3. Outcome Measures

The primary outcome of the study was percent ipsilesional and contralesional recruitment. Ipsilesional recruitment was defined as the percent increase in voxel activation surrounding the primary motor cortex. A 3 cm ROI was centered on the maximal activation coordinates. The number of voxels above the threshold was divided by the total number of voxels interrogated to determine a percent voxel above the threshold (%V_Thresh_). Percent recruitment was reported as the percent change (*Δ*%) in the %V_Thresh_ between the second and first scans. A patient had evidence of ipsilesional recruitment if the percent recruitment was greater than 0%.

Contralesional recruitment was analyzed using a laterality index (LI), described elsewhere [27]. In short, LI was determined by the bilateral activation during motor tasks in a 1 cm ROI centered on the maximal activation coordinates. LI was calculated as LI = (*v*_*i*_ − *v*_*c*_)/(*v*_*i*_ + *v*_*c*_), where *v*_*i*_ and *v*_*c*_ denote the number of voxels activated in the ipsilesional and contralesional regions, respectively. The change in LI (*Δ*LI) was the difference between the second scan and the first scan. A patient was defined as having contralesional recruitment if *Δ*LI was greater than 0.

Supplemental motor activation (SMA) was analyzed similar to ipsilesional recruitment as described above. A 1 cm ROI was centered on the maximal activation in the SMA region, along the midline and immediately anterior to the primary motor cortex. SMA recruitment was defined as the percent change in voxels above the threshold divided by the total voxels interrogated before and after surgery.

### 2.4. Statistical Analysis

Percent ipsilesional recruitment, contralesional recruitment, and SMA recruitment along with patient-specific characteristics were recorded and imported into a separate worksheet and analyzed using IBM SPSS Statistics for Windows, version 24.0 (IBM Corp., Armonk, N.Y., USA). Nonparametric Mann-Whitney *U* tests were used to compare factors and percent recruitment (Table 1). Correlation data for two continuous variables was computed using Spearman's Rank Order Correlation for ipsilesional and contralesional recruitment separately. For all analysis, statistical significance was set at *p* < 0.05, two tailed.

## 3. Results

Thirteen patients were included in our study (Figure 1).  Table 2 lists key patient- and tumor-specific parameters for each subject enrolled in the study. All patients (mean age: 50 ± 14) had a pathology-proven diagnosis of glioma. Timing between fMRI scans was an average of 15 months. Average tumor volume was 20.9, and the average growth rate of the tumors was 1.12 ± 1.23 cm^3^/month. The average distance from the tumor edge (as detected by FLAIR signal on MRI) to the center of the hand motor region was 3.9 ± 3.5 cm (Table 2).

Plasticity was quantified for each patient as either ipsilesional or contralesional with respect to the tumor location side. Ipsilesional plasticity was calculated as the percent increase in the number of voxels above the threshold in the ipsilateral hand motor cortex of the precentral gyrus at the second time point relative to the first. Thus, a positive number indicated the presence of ipsilesional plasticity. Contralateral plasticity was defined as a positive change in the laterality index at the second time point. Eight of the 13 patients had evidence of ipsilesional recruitment, and six of the 13 patients exhibited contralesional recruitment. Two patients had both patterns of recruitment, and only one patient had neither pattern.

**Figure 2:**
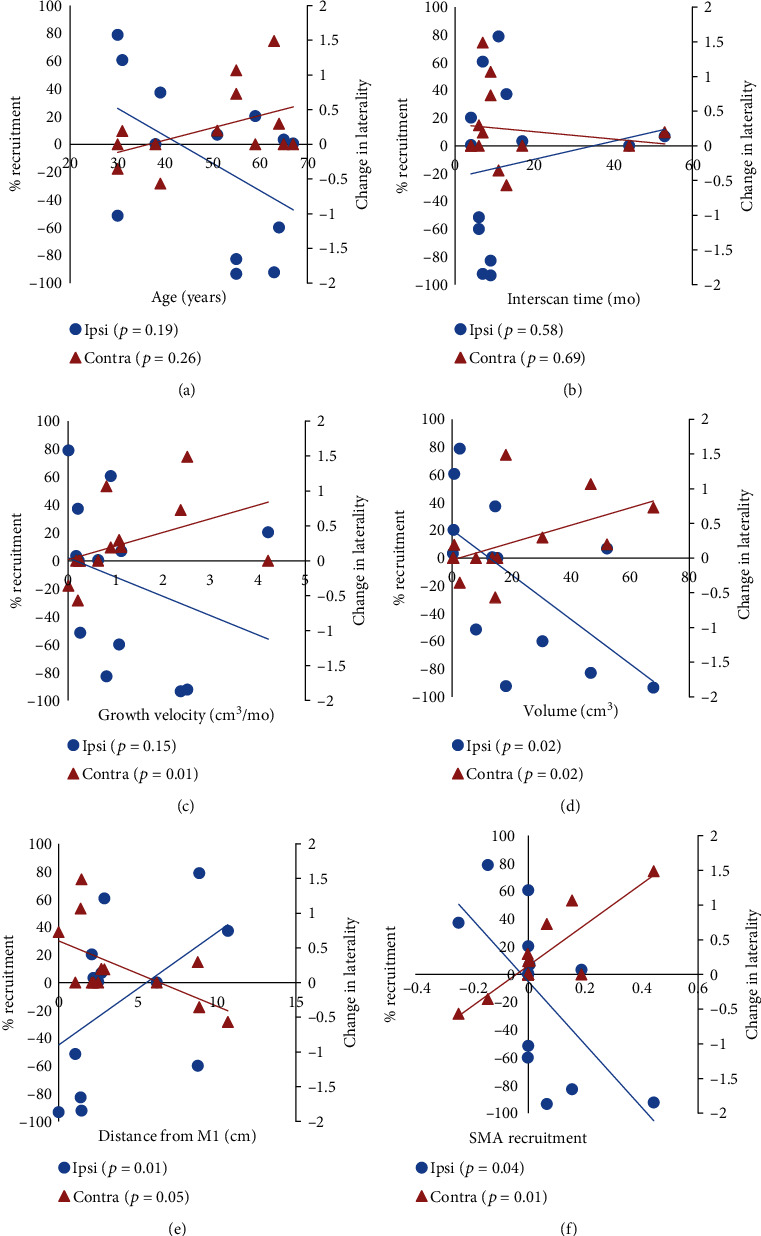
Correlation plots of ipsilesional and contralesional plasticity against patient-level factors. Ipsilesional recruitment was measured via percent recruitment (blue circles) and contralesional recruitment was measured via change in laterality (orange triangles) as computed from serial fMRI scans. Ipsilesional and contralesional graphs were placed on the same *x*-axis for efficiency. Mode of recruitment is plotted versus (a) age (years), (b) time between scans (months), (c) growth velocity (cm^3^/mo), (d) tumor volume (cm^3^), (e) distance from motor cortex (cm), and (f) degree of SMA recruitment (% change) from the first scan to the second scan. *p* values for each correlation are listed in each plot, with significant correlations (*p* ≤ 0.05) in bold.

**Figure 3:**
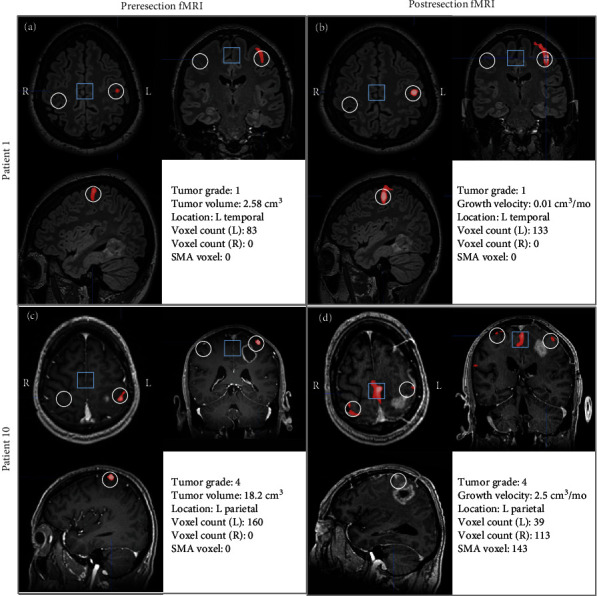
Examples of two modes of primary motor cortex plasticity. (a, b) Patient 1 had a slow growing (velocity = 0.01 cm^3^/mo) grade I primary brain tumor in the left temporal lobe (distant from primary motor cortex). Voxel-based analysis revealed recruitment on the ipsilesional (L) hand motor cortex but no recruitment in the contralesional (R) side (white circles). (c, d) Patient 10 had a rapidly growing (velocity = 2.5 cm^3^/mo) grade IV primary brain tumor in the left parietal lobe. Voxel-based analysis revealed decreased activation on the ipsilesional (L) side along with increased recruitment on the contralesional (R) side. This contralesional shift of function was accompanied by increased recruitment in the supplementary motor area (SMA, blue box). (a, c) represent images from each patient's initial fMRI while (b, d) represent images from each patient's second fMRI. For all images, red voxels represents activated areas during right-handed finger tapping with increasing brightness representing greater activation. White circles represent the region of interest that was analyzed for voxel counts. Blue squares represent region of interest for computing SMA activation counts.

Regression analysis revealed that both ipsilesional recruitment and contralesional recruitment were significantly correlated with two factors: tumor volume and distance between tumor edge and hand region of the primary motor cortex (M1) (Figure 2). Specifically, as tumor volume increased, ipsilesional recruitment decreased (*p* = 0.02) while contralesional recruitment increased (*p* = 0.02) (Figure 2(d)). Conversely, as the tumor was more distant from M1, ipsilesional recruitment increased (*p* = 0.01) while contralesional recruitment decreased (*p* = 0.05) (Figure 2(e)). Contralesional recruitment was positively correlated with tumor growth velocity (*p* = 0.01) (Figure 2(c)). Further, recruitment of the supplementary motor area (SMA) was associated with increased contralesional plasticity (*p* = 0.01) while reduction in SMA activity was correlated with decreased levels of ipsilateral plasticity (*p* = 0.04) (Figure 2(f)).  Figure 3 provides examples of ipsilesional and contralesional recruitment as a function of tumor and patient parameters.

Group-level analyses revealed a number of additional parameters that were significant with respect to ipsilesional and contralesional plasticity (Table 1). Ipsilesional recruitment was increased in patients with lesions with a total volume less than 16 cm^3^ (*p* = 0.02), tumors ≥ 2 cm from M1 (*p* = 0.01), and decreased SMA activation (*p* = 0.04). Significant contralesional recruitment was observed in patients age 50 or older (*p* = 0.04), had less than 10 months between scans (*p* = 0.05), tumor volume ≥ 16 cm^3^ (*p* = 0.003), growth velocities greater than 0.7 cm^3^/month (*p* = 0.01), tumors < 2 cm from M1 (*p* = 0.04), having a deficit on the first clinical exam (*p* = 0.05), and increased SMA activation (*p* = 0.01). No significant relationships were observed between the pattern of recruitment and a decrease of clinical function between the two exams (*p* = 0.08, *p* = 0.14).

## 4. Discussion

Little is known regarding the mechanisms of plasticity resulting from damage to the human primary motor cortex. In this retrospective case series, we found that plasticity was quite common with 12 of the 13 patients demonstrating at least one pattern of plasticity. Recruitment of brain tissue near the region of injury (ipsilesional plasticity) was observed in 10 of the 13 patients, and factors associated with this particular mode of plasticity included small tumors distant from the primary motor cortex. Recruitment of the primary motor cortex contralateral to the tumor location (contralesional plasticity) was seen in 8 of the 13 patients in our cohort and was associated with larger tumors near or in the primary motor cortex with higher growth velocity. In addition, contralesional recruitment was associated with increased activation of the supplemental motor area (SMA) within the posterior portion of the superior frontal gyrus, just anterior to the precentral sulcus. Finally, contralesional plasticity appears to be associated with shorter interscan times. These findings suggest that in response to accumulated injury in the human brain, there may be a trade-off between the two types of recruitment, with certain injury types favoring one type over the other based on patient/tumor conditions and timing after injury onset. Patients with smaller tumors that are further away from the primary motor cortex have the capacity to recruit neurons in or near the primary motor cortex on the same side as the injury. However, patients who are older and have larger, faster growing tumors that are closer to or directly invading the primary motor cortex lead to contralateral recruitment. Our findings are consistent with data from patients with ischemic strokes demonstrating motor cortex disinhibition and increased contralesional activity following both acute and chronic strokes [9, 28, 29]. Additionally, patients with clinical deficits on initial exam, greater areas of injury, or injuries within M1 show a more diffuse pattern of activation including increased contralesional activity [30, 31].

With respect to mechanisms of contralesional recruitment, prior studies have hypothesized that inhibitory fibers connect the two primary motor cortices, and thus, injury in one hemisphere could result in disinhibition of the analogous contralateral motor cortex [9, 15]. In support of this notion, application of GABA antagonists in rodents results in corticospinal tracts having bilateral terminations, and in fact, as many as 11% of axonal projections may never decussate in the spinal cord [16]. Together with our data, we speculate that aggressive, fast-growing tumors may overwhelm ipsilateral plasticity mechanisms from direct damage to the primary motor region and disruption of inhibitory crossing fibers projecting to the contralateral hemisphere. Once disinhibited, the contralesional motor cortex has increased activation allowing for compensation of the affected muscle groups through preexisting nondecussating spinal tracts. Our data also demonstrates that SMA activation is associated with contralesional recruitment, which is consistent with a prior study showing increased SMA activation in the context of functional recovery following frontal lobe surgery [32]. However, we recognize that BOLD signaling in fMRI is an indirect measure of neural activation and not the ideal method to testing neural mechanisms. Further prospective analyses investigating the neural mechanisms behind brain plasticity are warranted.

In patients with ischemic stroke, it has been shown that the degree of functional deficit is directly correlated with the amount of contralesional recruitment [33]. We hypothesized that patients with greater functional deficits on the clinical exam would exhibit more neural compensation as the brain tried to mitigate the effects of the damage. Although not statistically significant in our small patient cohort, patients with no clinical deficits showed a greater propensity towards ipsilesional recruitment while those with significant neurologic deficits tended to not recruit ipsilesionally (*p* = 0.06).

A few limitations of our study should be noted. First, the study was retrospective and had a limited sample size. Second, the fMRI scans were performed during the course of routine clinical care and not solely for research purposes, and thus, we had variability in follow-up times, patient effort among the scans, and MRI hardware. We acknowledge that patients with longer follow-up time would have a greater potential for plasticity. We attempted to standardize patient effort by excluding any patients who could complete the finger-tapping task. The change in MRI hardware only affected 2 patients, and given the relatively similar settings, we do not believe this influenced our overall conclusions substantially. Third, registration of patients' fMRI scans to standard MNI coordinates is challenging in our cohort given the significant structural changes. To minimize error, we masked the FLAIR signal and/or resection cavity for each image prior to registration. Additionally, we utilized linear transformation methods which have been shown to be accurate in normalization datasets that have high degrees of local mass effect, resection cavity distortion, or ventricular changes [23]. Finally, only patients who were fit enough to be considered for surgery were included, thus introducing the possibility of selecting for patients with high plasticity potential. It should also be noted that cutoff values in Table 1 represent appropriate values for our case series. Further work to establish generalizability of cutoff values should be explored.

In summary, through an innovative approach examining serial motor fMRI data in patients harboring brain lesions of varying size, location, and growth velocity, the current study suggests two patterns of neural recruitment in both near and distant brain regions following injury. Ultimately, these data may be informative for physicians designing individualized treatment and rehabilitation strategies to optimize functional recovery in patients with brain disorders by providing potential anatomic targets for therapy as well as optimal time windows for intervention.

## Figures and Tables

**Table 1:** Group level analysis for different factors affecting ipsilesional and contralesional recruitment. Ipsilesional recruitment and contralesional recruitment are reported as a group mean with standard error and standard deviations, respectively. Group differences were considered significant if *p* ≤ 0.05.

	Ipsilesional (S.E.)	*p* value	Contralesional (S.D.)	*p* value
Age (yrs)				
<50	25.1 (14.4)	0.079	-0.15 (0.09)	0.035
≥50	-37.2 (13.7)		0.47 (0.16)	
Interscan (mo)				
<10	-37.3 (16.0)	0.079	0.47 (0.16)	0.050
≥10	25.3 (9.3)		-0.15 (0.09)	
Velocity (cm^3^/mo)				
<0.7	11.4 (17.8)	0.285	-0.15 (0.10)	0.008
≥0.7	-34.4 (23.7)		0.57 (0.21)	
Volume (cm^3^)				
<16	18.7 (11.3)	0.019	-0.09 (0.07)	0.003
≥16	-64.3 (11.6)		0.75 (0.15)	
Distance (cm)				
<2	-80.0 (5.4)	0.009	0.82 (0.17)	0.039
≥2	16.4 (11.1)		-0.03 (0.08)	
Decrease in function				
No	5.4 (51.4)	0.077	0.05 (0.35)	0.139
Yes	-75.5 (21.3)		0.85 (0.77)	
Deficit on exam 1				
No	8.5 (46.0)	0.139	-0.03 (0.28)	0.047
Yes	-62.0 (55.1)		0.82 (0.63)	
SMA recruitment				
No	10.8 (13.7)	0.042	-0.05 (0.08)	0.014
Yes	-51.6 (14.4)		0.69 (0.17)	

**Table 2:** Patient characteristics. Characteristic information from patient cohort including age, hand dominance (L/R), time between scans (months), pathologic grade, growth rate (cm^3^/month), tumor volume at initial fMRI (cm^3^), distance from edge of tumor to primary motor cortex (cm), and NANO grade of the weakest limb (1^st^ exam, 2^nd^ exam).

Subject	Age (yrs)	Hand	Interscan (mo)	Grade	Velocity (cm^3^/mo)	Volume (cm^3^)	Distance (cm)	NANO grade
1	30	R	11	I	0.01	2.58	8.9	0, 0
2	55	R	9	IV	2.37	67.9	0	1, 1
3	51	L	53	II	1.12	52.3	2.7	0, 0
4	55	R	9	IV	0.81	46.8	1.4	1, 2
5	30	L	6	IV	0.25	8.1	1.06	0, 1
6	67	R	4	IV	0.63	13.6	2.5	0, 0
7	64	R	6	IV	1.07	30.5	8.8	0, 0
8	31	R	7	II	0.90	0.73	2.9	0, 0
9	39	R	13	IV	0.21	14.6	10.7	0, 0
10	63	L	7	IV	2.51	18.2	1.45	1, 2
11	59	R	4	IV	4.22	0.52	2.1	1, 1
12	65	R	17	IV	0.17	0.33	2.2	0, 0
13	38	R	44	III	0.23	15.3	6.2	0, 0
Avg	49.8		14.6	3.38	1.12	20.9	3.9	

## Data Availability

The datasets generated and used during the current study are available from the corresponding author on reasonable request.
